# Supplementary material for: Talin and kindlin use integrin tail allostery and direct binding to activate integrins
Source: Nat Struct Mol Biol. 2023 Dec 12;30(12):1913–24. doi: 10.1038/s41594-023-01139-9 (PMC10716038; doi:10.1038/s41594-023-01139-9)
Supplement: Supplementary file 1 — Reporting Summary [file 41594_2023_1139_MOESM1_ESM.pdf]

Corresponding author(s): Reinhard Fässler

Last updated by author(s): Jun 22, 2023

Reporting Summary

Nature Portfolio wishes to improve the reproducibility of the work that we publish. This form provides structure for consistency and transparency in reporting. For further information on Nature Portfolio policies, see our Editorial Policies and the Editorial Policy Checklist.

Statistics

For all statistical analyses, confirm that the following items are present in the figure legend, table legend, main text, or Methods section.

n/a	Confirmed	
☐	☒	The exact sample size (*n*) for each experimental group/condition, given as a discrete number and unit of measurement
☐	☒	A statement on whether measurements were taken from distinct samples or whether the same sample was measured repeatedly
☐	☒	The statistical test(s) used AND whether they are one- or two-sided<br>*Only common tests should be described solely by name; describe more complex techniques in the Methods section.*
☐	☒	A description of all covariates tested
☐	☒	A description of any assumptions or corrections, such as tests of normality and adjustment for multiple comparisons
☐	☒	A full description of the statistical parameters including central tendency (e.g. means) or other basic estimates (e.g. regression coefficient) AND variation (e.g. standard deviation) or associated estimates of uncertainty (e.g. confidence intervals)
☐	☒	For null hypothesis testing, the test statistic (e.g. *F*, *t*, *r*) with confidence intervals, effect sizes, degrees of freedom and *P* value noted<br>*Give P values as exact values whenever suitable.*
☒	☐	For Bayesian analysis, information on the choice of priors and Markov chain Monte Carlo settings
☒	☐	For hierarchical and complex designs, identification of the appropriate level for tests and full reporting of outcomes
☒	☐	Estimates of effect sizes (e.g. Cohen's *d*, Pearson's *r*), indicating how they were calculated

Our web collection on statistics for biologists contains articles on many of the points above.

Software and code

Policy information about availability of computer code

Data collection

NMR: Topspin 3.2-3.5 (Bruker)
MST: NT Control v2.1.33 (Nanotemper);
Flow Cytometry: FACSDiva Software Version 9.0 (BD);
CL-MS: Foundation 3.1SP7, Xcalibur 4.3 (Thermo Fisher Scientific);
SCFS: NanoWizard control software version 4.3.55 (JPK);
Microscopy: Auto 2 (Invitrogen), VisiView 4.0 (Visitron Systems GmbH);
Plate reader: SoftMax Pro 7.1 (Molecular Devices);
Thermal stability: PR.Therm Control Version 2.1.2 (Nanotemper);
Dynamic light scattering: Dynamics 8.0.0.89 (Wyatt);
CD spectroscopy: Spectra Manager Version 2.12.00 (Jasco);

Data analysis

NMR: Topspin 3.2-3.5 (Bruker), NMR Pipe (NIST IBBR), CcpNmr Analysis 2.5.2 (Skinner et al. J Biomol NMR 66 (2016) 111), TALOS-N (Yang Shen, and Ad Bax, J. Biomol. NMR, 56, 227-241(2013));
MST: MO Affinity Analysis v2.3 (Nanotemper);
Flow Cytometry: FlowJo 10.6.1, non-linear regression and statistical tests: OriginPro 2019b (OriginLab);
CL-MS: Proteome Discoverer (Thermo Fisher Scientific, version 2.5.0.400) with the XlinkX/PD nodes integrated (Klykov et al., 2018), Visualization: XMAS 1.1.1 plug-in (Scheltema lab) in ChimeraX 1.3 (UCSF)
SCFS: Data Processing version 4.3.55 (JPK). Statistical tests were performed using Prism (GraphPad Software - Version 8.4.3 (471));
Microscopy: ImageJ 1.49 (Wayne Rasband, NIH); focal adhesion analysis server (https://faas.bme.unc.edu/, Shawn Gomez lab), statistical tests: OriginPro 2019b (OriginLab);
Plate reader: SoftMax Pro 7.1 (Molecular Devices), statistical tests: OriginPro 2019b (OriginLab);

Thermal stability: PR.Therm Control Version 2.1.2 (Nanotemper);
Dynamic light scattering: Dynamics 8.0.0.89 (Wyatt)
CD spectroscopy: Spectra Manager Version 2.12.00 (Jasco);

For manuscripts utilizing custom algorithms or software that are central to the research but not yet described in published literature, software must be made available to editors and reviewers. We strongly encourage code deposition in a community repository (e.g. GitHub). See the Nature Portfolio guidelines for submitting code & software for further information.

Data

Policy information about availability of data

All manuscripts must include a data availability statement. This statement should provide the following information, where applicable:
- Accession codes, unique identifiers, or web links for publicly available datasets
- A description of any restrictions on data availability
- For clinical datasets or third party data, please ensure that the statement adheres to our policy

No data with mandated deposition was generated in this study. The data is available upon request.

Human research participants

Policy information about studies involving human research participants and Sex and Gender in Research.

Reporting on sex and gender	N/A
Population characteristics	N/A
Recruitment	N/A
Ethics oversight	N/A

Note that full information on the approval of the study protocol must also be provided in the manuscript.

Field-specific reporting

Please select the one below that is the best fit for your research. If you are not sure, read the appropriate sections before making your selection.

☒ Life sciences ☐ Behavioural & social sciences ☐ Ecological, evolutionary & environmental sciences

For a reference copy of the document with all sections, see nature.com/documents/nr-reporting-summary-flat.pdf

Life sciences study design

All studies must disclose on these points even when the disclosure is negative.

Sample size	No statistical method was used to predetermine the sample size. Key experiments were repeated at least three times to allow for a statistical analysis.
Data exclusions	Only data of insufficient quality was excluded. The quality criteria were: MST: According to manufacturer's recommendation, data points that vary by more than 20% fluorescence intensity compared to the average intensity of the other data points or that show aberrant MST traces, which might be a sign for aggregation, were excluded. FC-RDA: Titrations with insufficient signal-to-noise were not considered. DLS: Scans were filtered with 0.01 baseline value.
Replication	All cell culture experiments were performed at least thrice on three individual days. Key cell culture experiments were confirmed with a second cell line. Experiments with nanodiscs were repeated at least thrice with different nanodisc preparations. All affinity measurements were performed with at least two different protein batches. Key affinity measurements were performed by different operators. All replication experiments were successful, except for individual nanodisc preparations that could not be used for FC-RDA experiments due to insufficient loading of TM-CTs.
Randomization	The order of contact times in SCFS was randomized for every cell and condition. All other experiments were not randomized as the measurements were in equilibrium and timing was therefore not cruicial.
Blinding	Microscopic images for cell spreading were recorded automatically, whereas images for focal adhesion analysis were recorded by a different operator. Blinding in all other experiments was not relevant since all measurements were analyzed after data acquisition and were therefore not susceptible to experimenter bias.

Reporting for specific materials, systems and methods

We require information from authors about some types of materials, experimental systems and methods used in many studies. Here, indicate whether each material, system or method listed is relevant to your study. If you are not sure if a list item applies to your research, read the appropriate section before selecting a response.

Materials & experimental systems

n/a	Involved in the study
☐	☒ Antibodies
☐	☒ Eukaryotic cell lines
☒	☐ Palaeontology and archaeology
☒	☐ Animals and other organisms
☒	☐ Clinical data
☒	☐ Dual use research of concern

Methods

n/a	Involved in the study
☒	☐ ChIP-seq
☐	☒ Flow cytometry
☒	☐ MRI-based neuroimaging

Antibodies

Antibodies used	anti-β1 integrin (total level, biotinylated): eBioscience, 13-0291-80; anti-β1 integrin (9EG7, extended conformation): PharMingen, 550531; anti-β1 integrin PE: BioLegend, 102207; anti-β3 integrin PE: eBioscience, 12-0611; anti-αV integrin PE: BD, 551187; anti-α5 integrin PE: PharMingen, 557447; anti-GAPDH: Calbiochem, CB1001; Goat anti-mouse IgG HRP conjugate: BioRad, 1721011; anti-rat 647: Invitrogen, A21247; Streptavidin eFluor780: eBioscience, 47-4317-82; Rat IgG1 PE isotype control: PharMingen, 554685; Rat IgG2 PE isotype control: PharMingen, 555844; Hamster IgG PE isotype control: eBioscience, 1091682; anti-kindlin-2 (MAB2617): Merck Millipore; anti-talin-HRP: Santa Cruz, sc-365875; anti-talin: Sigma, T3287.
Validation	β1 integrin (clone eBioHMb1-1, 13-0291-80, hamster, eBioscience, Wu et al., Bone, 2008. FC); β1 integrin (clone 9EG7, 550531, rat, PharMingen, Lenter et al., PNAS, 1993. FC); β1 integrin (clone HMβ1-1, 102207, hamster, BioLegend, Noto K, et al., Int Immunol, 1995. FC).; β3 integrin (clone 2C9.G3, 12-0611, hamster, eBioscience, Treese et al., Cytometry Part A, 2008. FC); αV integrin (clone RMV-7, 551187, rat, BD, Bader et al., Cell, 1998. FC); α5 integrin (clone 5H10-27, 557447, rat, PharMingen, Kharbili et al, Oncotarget. 2017. FC); GAPDH (clone 6C5, CB1001, mouse, Calbiochem, Gagarin et al., J. Mol. Cell. Card., 2005. WB); kindlin-2 (MAB2617, mouse, Merck Millipore, Theodosiou et al, eLife 2016. WB); talin-1 (T3287, Sigma, Theodosiou et al, eLife 2016; WB).

Eukaryotic cell lines

Policy information about cell lines and Sex and Gender in Research

Cell line source(s)	Mouse TLN1/TLN2/KIND1/KIND2 quadruple floxed fibroblast: generated in the lab<br>Mouse TLN1/TLN2 double floxed fibroblast: generated in the lab<br>Mouse ITGB1 knock-out fibroblasts: generated in the lab
Authentication	Mouse TLN1/TLN2/KIND1/KIND2 quadruple floxed fibroblasts were generated in our lab from transgenic mice and previously described in J Cell Biol, (2017) 216 (11): 3785.<br>Mouse TLN1/TLN2 double floxed fibroblasts were generated in our lab from transgenic mice and previously described in eLife (2016) 5:e10130.<br>Mouse ITGB1 knock-out fibroblasts were generated in our lab from transgenic mice and previously described in J Invest Dermatol (2013) 133, 2722.
Mycoplasma contamination	All cell lines were regularly tested negative for mycoplasma contamination.
Commonly misidentified lines<br>(See ICLAC register)	No commonly misidentified cell line was used in this study.

Flow Cytometry

Plots

Confirm that:

☒ The axis labels state the marker and fluorochrome used (e.g. CD4-FITC).

☒ The axis scales are clearly visible. Include numbers along axes only for bottom left plot of group (a 'group' is an analysis of identical markers).

☒ All plots are contour plots with outliers or pseudocolor plots.

☒ A numerical value for number of cells or percentage (with statistics) is provided.

Methodology

Sample preparation	The adhesive cell lines were detached from the culture plates with 500 µL trypsin and EDTA in PBS, trypsin was neutralized with 500 µL DMEM supplemented with 10% FBS, transferred into 4 mL DMEM supplemented with 10% FBS and split in different FACS tubes for antibody staining after washing twice with PBS.
Instrument	BD LSRFortessa X-20
Software	FACS data was recorded using: BD FACSDiva Software Version 9.0<br>FACS data was analyzed using: FlowJo 10.6.1
Cell population abundance	Defined cell lines were used.
Gating strategy	Cells were gated on the major cell population using an "auto gate" in FlowJo, that included approx. 70-80% of the recorded 10000 - 20000 events.

☒ Tick this box to confirm that a figure exemplifying the gating strategy is provided in the Supplementary Information.
