# Supplementary figures and images for: Talin and kindlin use integrin tail allostery and direct binding to activate integrins
Source: Nat Struct Mol Biol. 2023 Dec 12;30(12):1913–24. doi: 10.1038/s41594-023-01139-9 (PMC10716038; doi:10.1038/s41594-023-01139-9)

## Slide 1
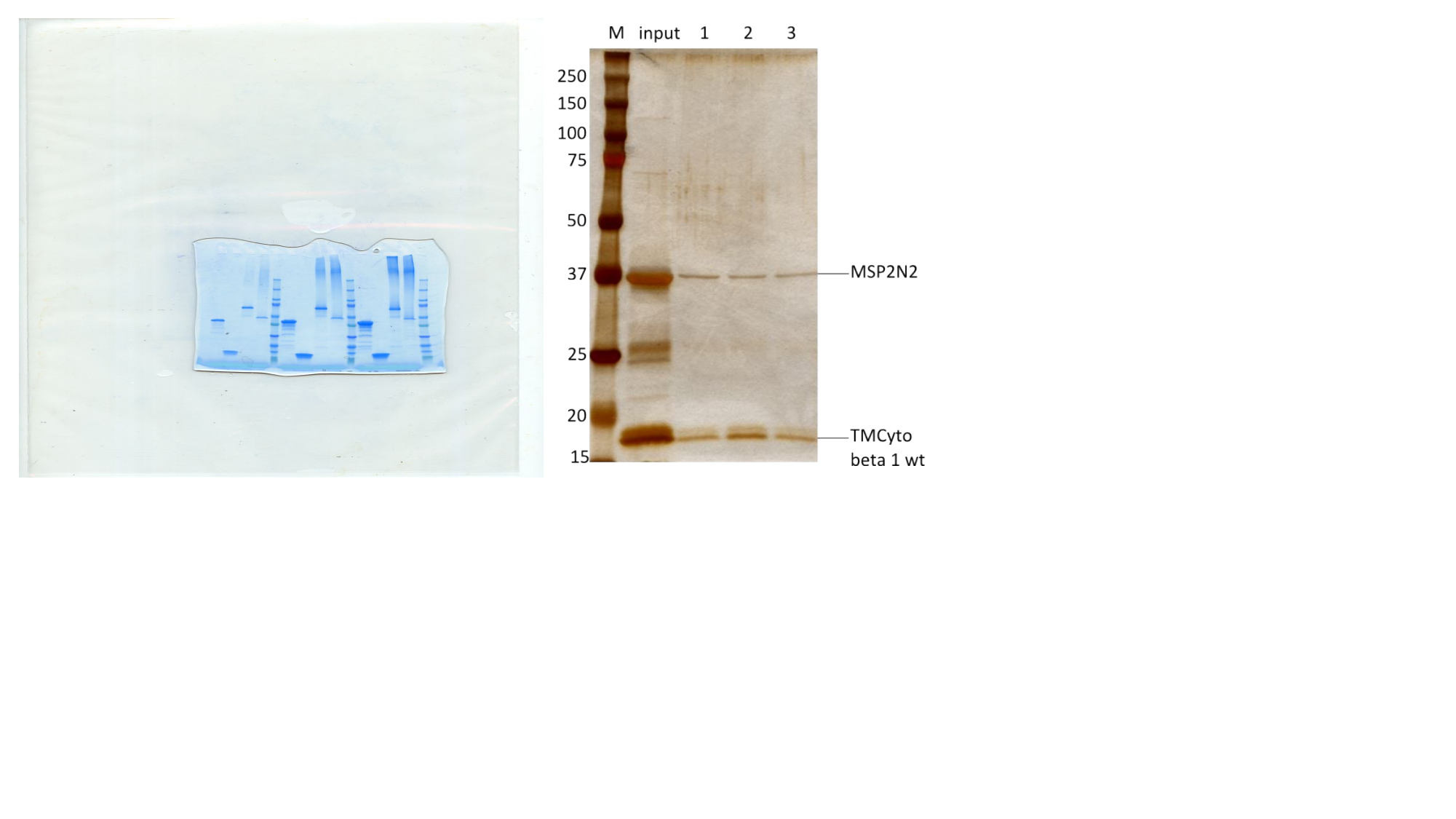

Supplement: Supplementary file 9 — Uncropped gel images of gels shown in Extended Data Fig. 1a. [file 41594_2023_1139_MOESM9_ESM.pptx]
